# Network pharmacological analysis on the mechanism of Linggui Zhugan decoction for nonalcoholic fatty liver disease

**DOI:** 10.1097/MD.0000000000037281

**Published:** 2024-03-08

**Authors:** Songlin Gao, Liuting Wei, Yan Qin, Peng Zhang, Tingwei Quan, Fei Liang, Guihua Huang

**Affiliations:** a Graduate School of Guangxi University of Chinese Medicine, Nanning, Guangxi, China; b Department of Nephrology, Liuzhou Traditional Chinese Medicine Hospital, Liuzhou, Guangxi, China; c Department of Spleen and Stomach Liver Diseases, The First Affiliated Hospital of Guangxi University of Chinese Medicine, Nanning, Guangxi, China.

**Keywords:** Linggui Zhugan decoction, mechanism of action, molecular docking, network pharmacology, nonalcoholic fatty liver disease

## Abstract

Nonalcoholic fatty liver disease (NAFLD), represents a chronic progressive disease that imposes a significant burden on patients and the healthcare system. Linggui Zhugan decoction (LGZGD) plays a substantial role in treating NAFLD, but its exact molecular mechanism is unknown. Using network pharmacology, this study aimed to investigate the mechanism of action of LGZGD in treating NAFLD. Active ingredients and targets were identified through the integration of data from the TCMSP, GEO, GeneCards, and OMIM databases. Cytoscape 3.9.1 software, in conjunction with the STRING platform, was employed to construct network diagrams and screen core targets. The enrichment analysis of gene ontology and the Kyoto Encyclopedia of Genes and Genomes pathways were conducted by using the R. Molecular docking of the active ingredients and core targets was performed with AutoDock Vina software. We obtained 93 and 112 active ingredients and potential targets using the bioinformatic analysis of LGZGD in treating NAFLD. The primary ingredients of LGZGD included quercetin, kaempferol, and naringenin. The core targets were identified AKT1, MYC, HSP90AA1, HIF1A, ESR1, TP53, and STAT3. Gene ontology function enrichment analysis revealed associations with responses to nutrient and oxygen levels, nuclear receptor activity, and ligand-activated transcription factor activity. Kyoto Encyclopedia of Genes and Genomes signaling pathway analysis implicated the involvement of the PI3K-Akt, IL-17, TNF, Th17 cell differentiation, HIF-1, and TLR signaling pathways. Molecular docking studies indicated strong binding affinities between active ingredients and targets. LGZGD intervenes in NAFLD through a multi-ingredient, multi-target, and multi-pathway approach. Treatment with LGZGD can improve insulin resistance, oxidative stress, inflammation, and lipid metabolism associated with NAFLD.

## 1. Introduction

Nonalcoholic fatty liver disease (NAFLD) is a liver disorder characterized by hepatic steatosis exceeding 5%, without secondary causes.^[1]^ NAFLD prevalence is rising worldwide, with a prevalence of 30% in China and 25% in the world.^[2,3]^ NAFLD is a major cause of hepatic sclerosis, liver cancer, and other liver diseases, burdening patients and the health system.^[4–6]^ A recent global burden of disease study reveals that NAFLD causes a heavy burden in Asia, the Middle East, and North Africa, accounting for about half of the global burden of hepatic sclerosis and hepatoma caused by NAFLD.^[7]^ No specific pharmacological treatment is available for NAFLD. The primary methods include a low-calorie diet, exercise therapy, and treatment of its complications; however, patients’ compliance is poor, and the curative effect is limited.^[8]^ To effectively address NAFLD, the development of targeted pharmaceuticals is imperative to enhance treatment options.

In the treatment of chronic diseases, traditional Chinese medicine (TCM) presents significant advantages, particularly in the context of liver diseases. TCM employs tailored treatments based on specific diagnoses for different diseases.^[9,10]^ NAFLD is classified under the TCM category of “Xie-Tong,” “Gan-Pi,” “Fei-Qi,” and “Ji-Ju.” TCM has been widely recognized for its efficacy in preventing and treating NAFLD, offering precise therapeutic effects with minimal adverse reactions.^[11]^ Hui D^[12]^ reported that a spleen-strengthening and liver-draining formula demonstrated the capacity to reduce AST, ALT, and liver fat content in NAFLD patients by regulating intestinal flora. Shuganzhi Tablet effectively treated NAFLD, while hesperidin, polydatin, and naringin may be its most effective components.^[13]^

Linggui Zhugan decoction (LGZGD), derived from the Chinese medical text “Treatise on Cold and Miscellaneous Diseases,” is composed of Fu Ling (*Poria cocos*), Gui Zhi (*Cinnamomi ramulus*), Bai Zhu (*Atractylodes macrocephala*), and Gan Cao (*Glycyrrhiza uralensis*). Fu Ling, the primary drug in this prescription, invigorates the middle energizer, removes dampness, and promotes diuresis. Gui Zhi, functioning as an assistant, contributes by warming the spleen yang and enhancing diuresis. Bai Zhu has the functions of invigorating the spleen and Qi, drying dampness, and diuresis. Gan Cao has the curative effect of Tonifying Qi and harmonizing various drugs. Combining the 4 drugs has the function of Warming Yang and promoting diuresis. Currently, the pharmacological research of LGZGD primarily involves antioxidant, anti-inflammatory, regulation of lipid metabolism, insulin resistance, and liquid metabolism.^[14]^ High-quality articles exploring the mechanism of the efficacy of LGZGD in treating NAFLD remain scarce, despite supportive findings from clinical observational studies.^[15,16]^ Network pharmacology is a new technology that can systematically study the possible mechanism of drugs on diseases by constructing the interaction network of “drugs-diseases-targets-pathways” and provide new insights into how TCM treats diseases. We aim to study the effective components, targets, biological processes (BPs), and pathways of LGZGD in treating NAFLD using network pharmacological methods to provide new ideas for further study.

## 2. Materials and methods

### 2.1. Screening of LGZGD’s active ingredients and targets

The TCM systematic pharmacology database (TCMSP, https://old.tcmsp-e.com/tcmsp.php) was used to retrieve each TCM in LGZGD. The LGZGD active ingredients were screened form oral bioavailability ≥ 30% and drug-like ≥ 0.18. Then, the “Related Targets” option was selected to find all prediction targets associated with each active ingredient. All the screened targets were imported into UniProt (https://www.uniprot.org) database because of a nonstandard problem with searching targets in TCMSP database. The obtained target name was transformed into the target protein’s gene name. Simultaneously, the input target protein name was called the restricted species “*Homo sapiens*,” so all protein names were corrected.

### 2.2. Screening of NAFLD disease-related targets

A search for the keyword “nonalcoholic fatty liver disease” was conducted in Gene Expression Omnibus (GEO, https://www.ncbi.nlm.nih.gov/), GeneCards (https://www.genecards.org/), and Online Mendelian Inheritance in Man (OMIM, https://omim.org/). The results of search in the 3 databases were summarized and de-duplicated to identify NAFLD disease-related target. The NAFLD (GSE89632) expression profile was downloaded from the GEO. The GSE89632 dataset included 20 NAFLD samples and 24 healthy samples on the GPL14951 platform. The differentially expressed genes (DEGS) were obtained under |logFC| > 1 and P.adj < 0.05 and displayed as the heat map.

### 2.3. TCM-active ingredients-potential targets network construction

The common target genes shared between LGZGD and NAFLD were identified using the “Venn” R package, signifying potential treatment targets. Subsequently, to construct a comprehensive network depicting the relationship among “TCM-active ingredients-potential targets,” we generated network and type attribute files. These files were then imported into Cytoscape 3.9.1 software. The degree value of active ingredients was calculated using the “Network Analyzer” tool within Cytoscape 3.9.1 software.

### 2.4. Protein–protein interaction (PPI) network graph construction

The common targets were entered into the STRING database (https://string-db.org/) to construct the PPI network under the conditions of species selection “Homo sapiens,” “hide disconnected nodes in the network,” and “highest confidence (>0.90).” For further analysis, the “.TAV” file format of the PPI network information is uploaded to Cytoscape 3.9.1 software. Cytoscape’s CytoNCA tool was used for topology analysis to screen the core targets according to the ranking of the values of Degree, Betweenness centrality, Closeness centrality, Eigenvector centrality, Network centrality, and Local Average Connectivity-based method.

### 2.5. Gene ontology (GO) and Kyoto Encyclopedia of Genes and Genomes (KEGG) enrichment analysis

R (version 4.2.2) package “clusterProfiler” was used to analyze the common targets, including GO and KEGG enrichment analysis.

### 2.6. Molecular docking

This study investigated molecular docking for analyzing the characteristics of receptors and the formation of molecular complexes between receptors and small drug molecules via computer space and energy matching and to predict the operation process of complex structures. The ID and 3D model structure of the protein corresponding to core target, as a macromolecular receptor, was retrieved from the PDB database (http://www.rcsb.org/). Then, the water molecular structure and original small molecule ligands of the core target protein were removed using PyMOL 2.4.1 software. From PubChem database (https://pubchem.ncbi.nlm.nih.gov/), the 2D structure of the core active ingredient was downloaded as a small molecule ligand. The 2D structure of the active ingredient was added to Chem3D software, converted into a 3D structure that optimizes the minimized energy, and exported as a “.Mol2” format. Protein and active ingredients were pretreated using AutodockTools 1.5.6 and converted to “.pdbqt” format, and then they were docked using Autodockvina 1.1.2. Display of docking results was performed with PyMOL software.

## 3. Results

### 3.1. LGZGD active ingredient and target information

We obtained 7 active ingredients from Bai Zhu, 15 from Fu Ling, 92 from Gan Cao, and 7 from Gui Zhi. Among them, sitosterol is the common active ingredient from Gan Cao and Gui Zhi, so LGZGD has 120 active ingredients. We retrieved 249 targets corresponding to 120 active ingredients of LGZGD from the TCMSP database. After combined data deduplication and target protein name correction using the UniProt database, 227 active targets were included, corresponding to 103 active ingredients.

### 3.2. NAFLD disease target data

**Figure 1.**
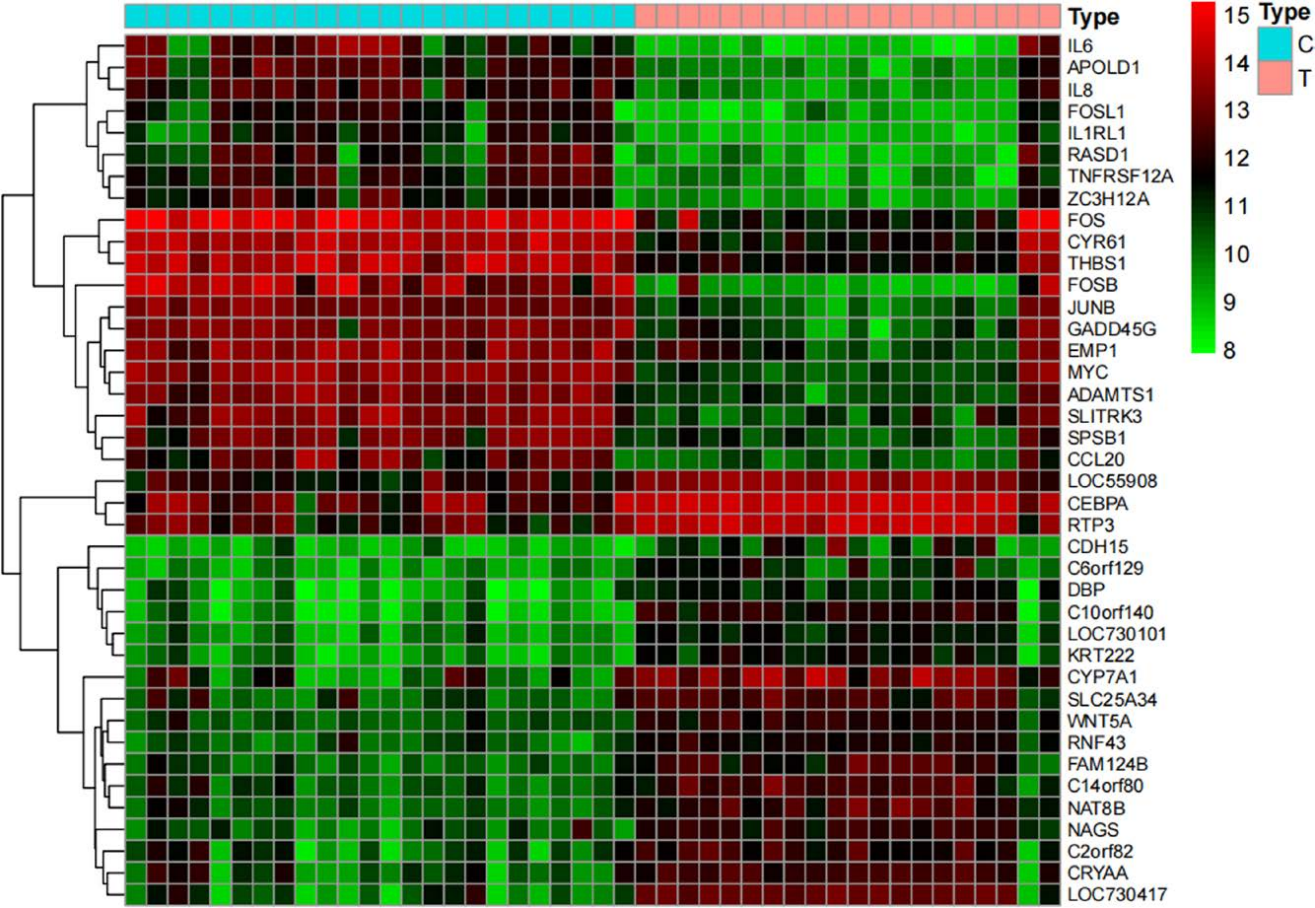
Heat map of NAFLD differentially expressed genes. NAFLD = nonalcoholic fatty liver disease.

**Figure 2.**
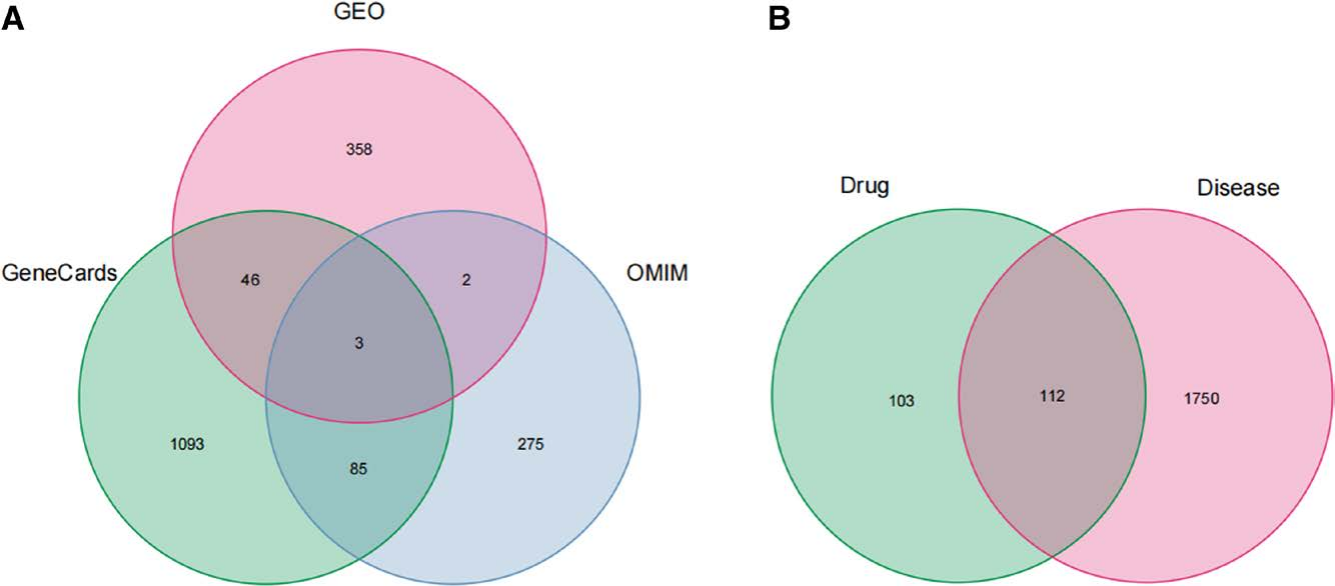
(A) Veen plots of NAFLD-related targets obtained based on GeneCards, OMIM, GEO databases, (B) Common targets of LGZGD and NAFLD. LGZGD = Linggui Zhugan decoction, NAFLD = nonalcoholic fatty liver disease.

We obtained 409 DEGS, including 135 up-regulated, from the GSE89632 dataset (Fig. 1). Additionally, 1227 and 365 disease targets were obtained in Genecards and OMIM databases, respectively. After the 3 database results were combined and de-duplicated, 1862 NAFLD disease targets were obtained (Fig. 2A).

### 3.3. TCM-active ingredients-potential targets network construction

**Figure 3.**
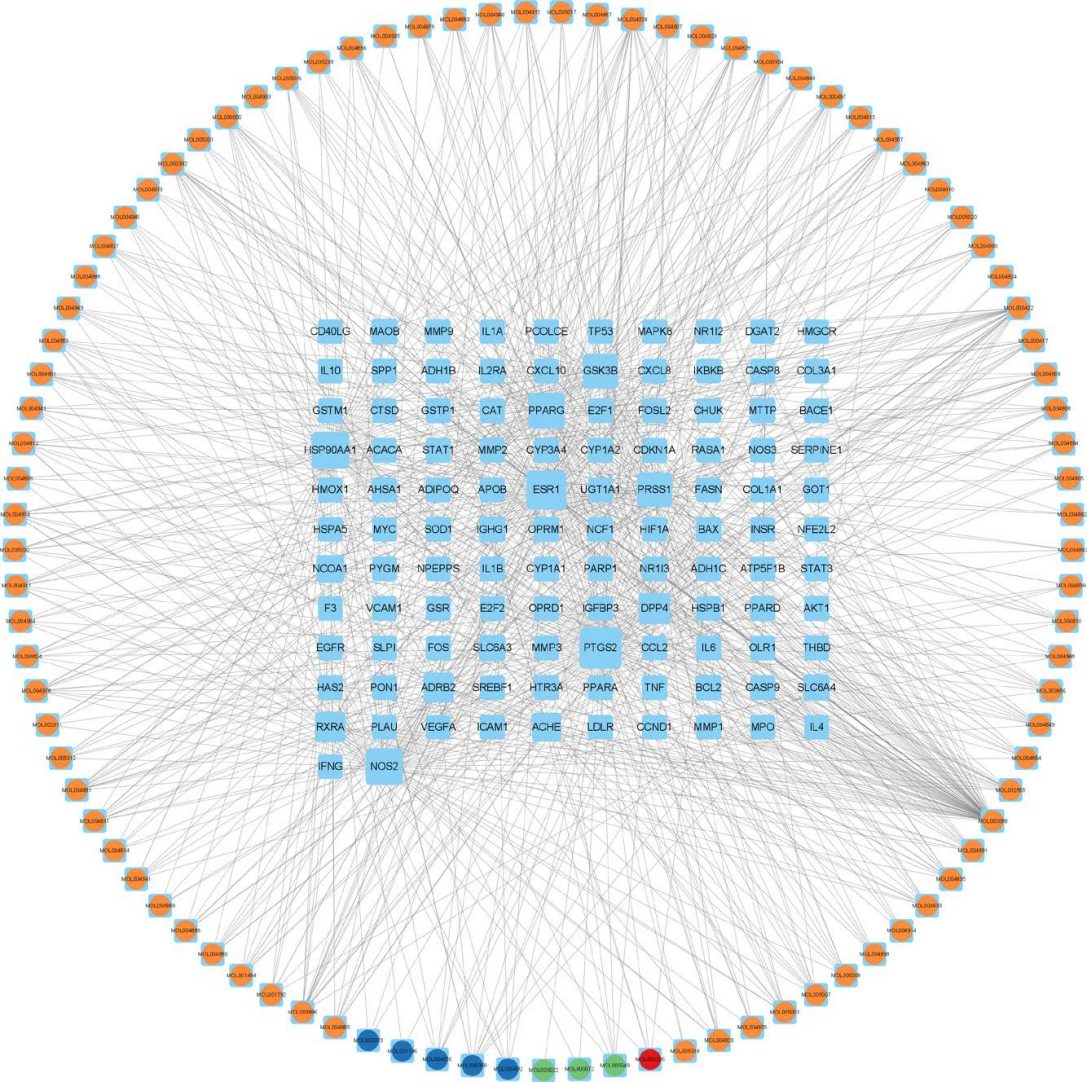
TCM-active ingredients-potential targets network. The circle in the network diagram represents TCM active ingredients, different colors represent TCM sources (orange represents Gan Cao, red represents Fu Ling, green represents Bai Zhu, blue represents Gui Zhi), and sky blue rectangular nodes represent potential targets. TCM = traditional Chinese medicine.

LGZGD exhibits 112 potential targets associated with 93 active ingredients in the treatment of NAFLD, as determined using R language software (4.2.2) (Fig. 2B). Then, the targets and ingredients were uploaded to Cytoscape 3.9.1 software for building a comprehensive “TCM-active ingredients-potential targets” network (Fig. 3). The circle in the network diagram represents TCM active ingredients, different colors represent TCM sources (orange represents Gan Cao, red represents Fu Ling, green represents Bai Zhu, blue represents Gui Zhi), and sky blue rectangular nodes represent potential targets. Nodes with higher degree values may signify key ingredients or targets of LGZGD in the treatment of NAFLD. The Network Analyzer results indicated that the top 3 active ingredients of the degree value are quercetin, kaempferol, and naringenin, which may be LGZGD’s key ingredients for treating NAFLD (Table 1).

**Table 1:** Core active ingredients of LGZGD in treating NAFLD.

Molecule ID	Molecule name	OB (%)	DL	Degree
MOL000098	Quercetin	46.43	0.28	78
MOL000422	Kaempferol	41.88	0.24	29
MOL004328	Naringenin	59.29	0.21	20

DL = drug like, LGZGD = Linggui Zhugan decoction, NAFLD = nonalcoholic fatty liver disease, OB = oral bioavailability

### 3.4. Construction and analysis of the PPI network

**Figure 4.**
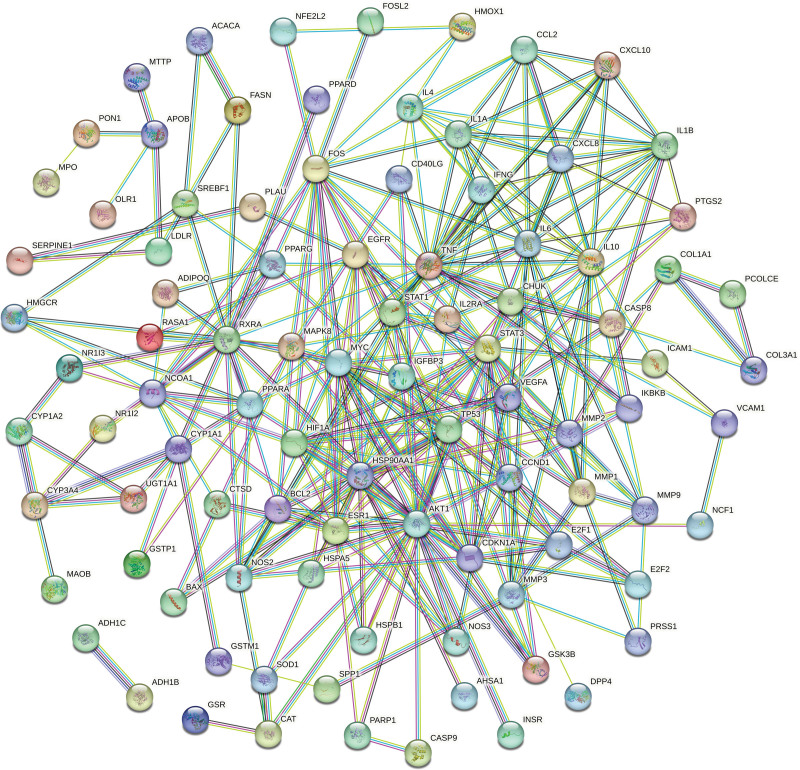
The PPI network of LGZGD and NAFLD targets. LGZGD = Linggui Zhugan decoction, NAFLD = nonalcoholic fatty liver disease, PPI = protein–protein interaction.

**Figure 5.**
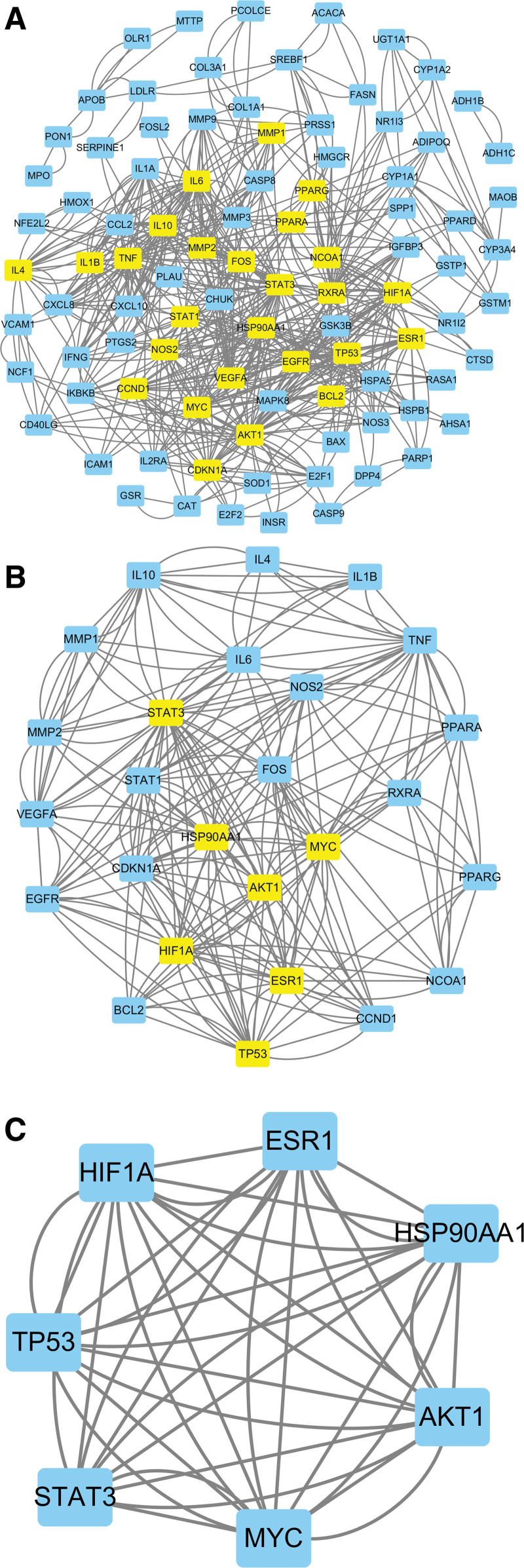
Screening process for core targets of LGZGD in treating NAFLD. (A) Filtered for the first time core network diagram, (B) filtered for the second time core network diagram, (C) streamlined core network, LGZGD = Linggui Zhugan decoction, NAFLD = nonalcoholic fatty liver disease.

The common targets were imported into the STRING database to create the PPI network diagram of potential targets of LGZGD for treating NAFLD (Fig. 4). The values of Degree, Betweenness centrality, Closeness centrality, Eigenvector centrality, Network centrality, and Local Average Connectivity-based method of the target were analyzed using the CytoNCA plug-in. After the first screening with these 6 values greater than the median, the second screening was conducted according to the same screening conditions. The core target was obtained via 2 screenings (Fig. 5). The core targets are serine/threonine kinase 1 (AKT1), myelocytomatosis (MYC), heat shock protein 90 alpha family class A member 1 (HSP90AA1), hypoxia-inducible factor-1alpha (HIF1A), estrogen receptor 1 (ESR1), cell tumor antigen p53 (TP53), and signal transducer and activator of transcription 3 (STAT3) (Table 2).

**Table 2:** Seven core targets of LGZGD in treating NAFLD.

Gene symbol	Uniprot ID	Protein name	Degree	Betweenness centrality	Closeness centrality	Eigenvector centrality	Network centrality	Local average connectivity-based method
AKT1	P31749	RAC-alpha serine/threonine-protein kinase	50	1308.320371	0.242819843	0.236547276	23.63724051	8.8
STAT3	P40763	Signal transducer and activator of transcription 3	50	871.5163929	0.236641221	0.318139493	29.04560658	13.44
TP53	P04637	Cellular tumor antigen p53	42	772.939031	0.2325	0.205045283	19.09237689	8.380952381
HSP90AA1	P07900	Heat shock protein HSP 90-alpha	40	498.9538806	0.233082707	0.235257775	19.86440742	10.6
MYC	P01106	Myc proto-oncogene protein	30	236.2699238	0.238461538	0.21578081	17.56505863	13.06666667
ESR1	P03372	Estrogen receptor	30	333.942824	0.2325	0.185871005	16.49279282	10.66666667
HIF1A	Q16665	Hypoxia-inducible factor 1-alpha	26	142.1953709	0.231343284	0.193065152	15.19762304	12.92307692

AKT1 = serine/threonine kinase 1; ESR1 = estrogen receptor 1; HIF1A = hypoxia-inducible factor-1alpha; HSP90AA1 = heat shock protein 90 alpha family class A member 1; MYC = myelocytomatosis; STAT3 = signal transducer and activator of transcription 3; TP53 = cell tumor antigen p53.

### 3.5. Enrichment analysis

**Figure 6.**
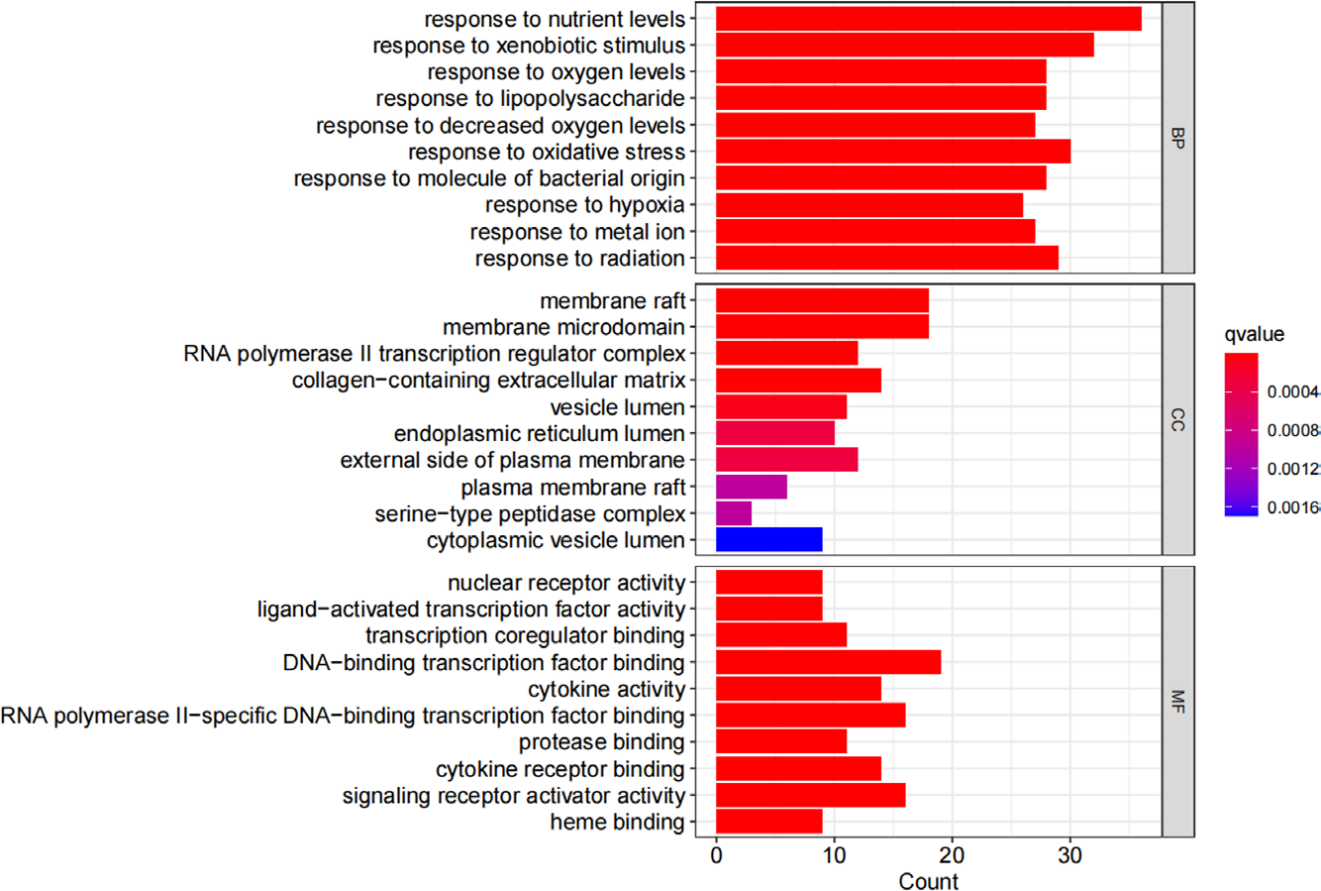
GO function enrichment analysis of targets of LGZGD in treating NAFLD. BP = biological process, CC = cellular component, GO = gene ontology, MF = molecular function.

The GO analysis presented that 2359 items were screened (*P* < .05). After ranking by *P*-value, the top 10 items for BP, cellular component, and molecular function were filtered to create a bar chart (Fig. 6 and Table 3). BP primarily involves the response to nutrient levels, xenobiotic stimulus, oxygen levels, lipopolysaccharide, decreased oxygen levels, oxidative stress, a molecule of bacterial origin, hypoxia, metal ion, and radiation. Cellular component was primarily enriched in membrane raft, membrane microdomain, RNA polymerase II transcription regulator complex, collagen-containing extracellular matrix, vesicle lumen, endoplasmic reticulum lumen, external side of the plasma membrane, plasma membrane raft, serine-type peptidase complex, and cytoplasmic vesicle lumen. Molecular function primarily included nuclear receptor activity, ligand-activated transcription factor activity, transcription coregulatory factor binding, DNA-binding transcription factor binding, cytokine activity, RNA polymerase II-specific DNA-binding transcription factor binding, protease binding, cytokine receptor binding, signaling receptor activator activity, and heme binding.

**Table 3:** The top 10 GO enrichment items of targets of LGZGD in treating NAFLD.

ID	Term	Category
GO:0031667	Response to nutrient levels	BP
GO:0009410	Response to xenobiotic stimulus	BP
GO:0070482	Response to oxygen levels	BP
GO:0032496	Response to lipopolysaccharide	BP
GO:0036293	Response to decreased oxygen levels	BP
GO:0006979	Response to oxidative stress	BP
GO:0002237	Response to molecule of bacterial origin	BP
GO:0001666	Response to hypoxia	BP
GO:0010038	Response to metal ion	BP
GO:0009314	Response to radiation	BP
GO:0045121	Membrane raft	CC
GO:0098857	Membrane microdomain	CC
GO:0090575	RNA polymerase II transcription regulator complex	CC
GO:0062023	Collagen-containing extracellular matrix	CC
GO:0031983	Vesicle lumen	CC
GO:0005788	Endoplasmic reticulum lumen	CC
GO:0009897	External side of plasma membrane	CC
GO:0044853	Plasma membrane raft	CC
GO:1905286	Serine-type peptidase complex	CC
GO:0060205	Cytoplasmic vesicle lumen	CC
GO:0004879	Nuclear receptor activity	MF
GO:0098531	Ligand-activated transcription factor activity	MF
GO:0001221	Transcription coregulator binding	MF
GO:0140297	DNA-binding transcription factor binding	MF
GO:0005125	Cytokine activity	MF
GO:0061629	RNA polymerase II-specific DNA-binding transcription factor binding	MF
GO:0002020	Protease binding	MF
GO:0005126	Cytokine receptor binding	MF
GO:0030546	Signaling receptor activator activity	MF
GO:0020037	Heme binding	MF

BP = biological process, CC = cellular component, GO = gene ontology, MF = molecular function.

**Figure 7.**
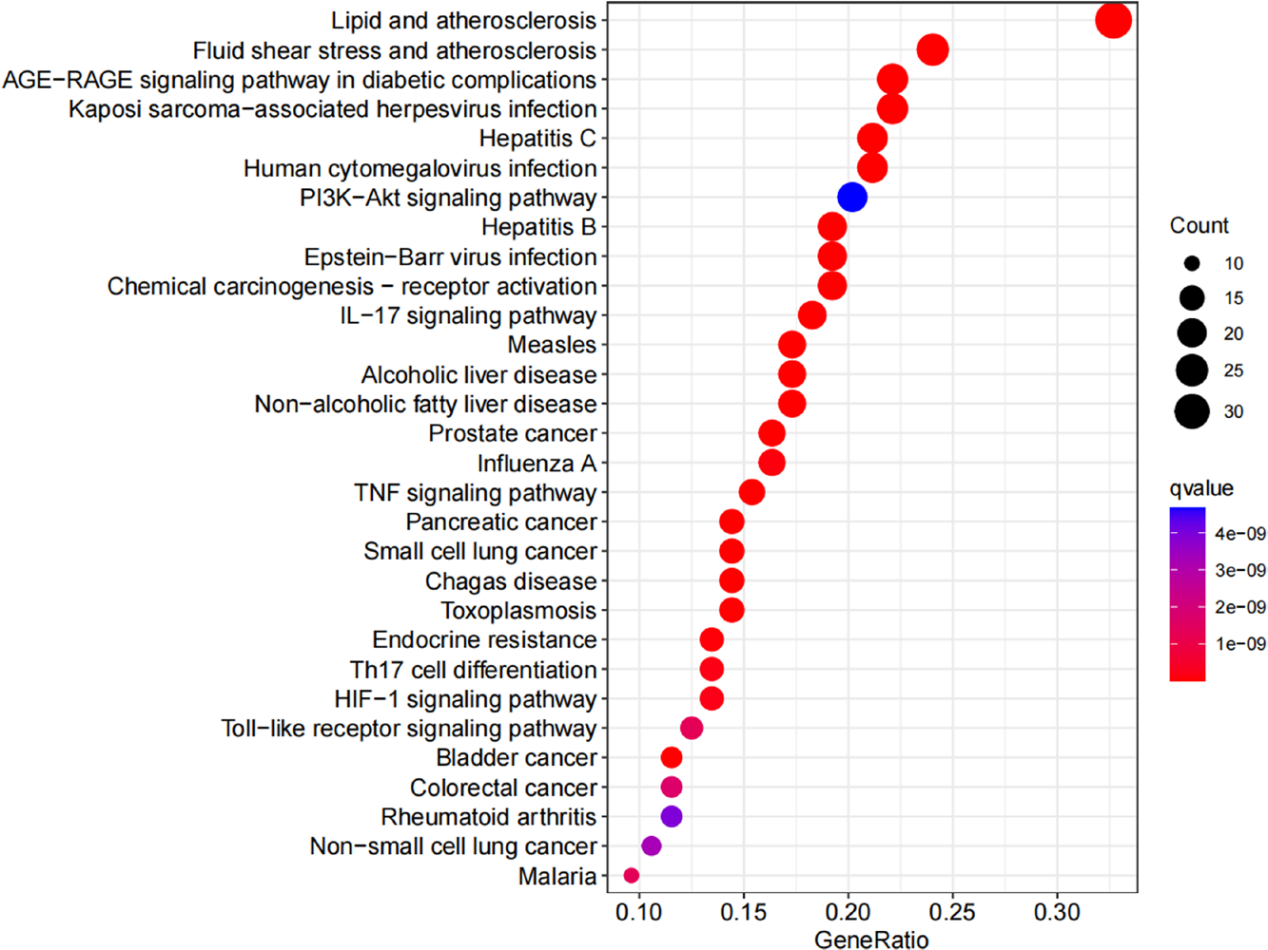
KEGG pathway enrichment analysis of targets of LGZGD in treating NAFLD. KEGG = Kyoto encyclopedia of genes and genomes, LGZGD = Linggui Zhugan decoction, NAFLD = nonalcoholic fatty liver disease.

KEGG data analysis obtained 158 related pathways (*P* < .05), including PI3K/Akt, IL-17, TNF, Th17 cell differentiation, HIF-1, and TLR signaling pathway (Fig. 7 and Table 4).

**Table 4:** The top 30 KEGG enrichment items of targets of LGZGD in treating NAFLD.

ID	Term	*P* value
hsa05417	Lipid and atherosclerosis	3.51E−29
hsa04933	AGE-RAGE signaling pathway in diabetic complications	1.26E−23
hsa05418	Fluid shear stress and atherosclerosis	8.28E−23
hsa04657	IL-17 signaling pathway	1.83E−18
hsa05160	Hepatitis C	1.15E−17
hsa05167	Kaposi sarcoma-associated herpesvirus infection	8.66E−17
hsa05215	Prostate cancer	1.69E−15
hsa05161	Hepatitis B	5.08E−15
hsa05212	Pancreatic cancer	1.36E−14
hsa05163	Human cytomegalovirus infection	2.66E−14
hsa05219	Bladder cancer	4.04E−14
hsa05162	Measles	5.79E−14
hsa04936	Alcoholic liver disease	8.46E−14
hsa05222	Small cell lung cancer	2.67E−13
hsa05169	Epstein-Barr virus infection	3.65E−13
hsa04932	Nonalcoholic fatty liver disease	3.97E−13
hsa04668	TNF signaling pathway	4.55E−13
hsa05207	Chemical carcinogenesis - receptor activation	9.12E−13
hsa05142	Chagas disease	1.28E−12
hsa05145	Toxoplasmosis	5.23E−12
hsa01522	Endocrine resistance	1.15E−11
hsa05164	Influenza A	2.41E−11
hsa04659	Th17 cell differentiation	4.43E−11
hsa04066	HIF-1 signaling pathway	5.04E−11
hsa04620	Toll-like receptor signaling pathway	3.66E−10
hsa05144	Malaria	3.76E−10
hsa05210	Colorectal cancer	4.94E−10
hsa05223	Non-small cell lung cancer	1.00E−09
hsa05323	Rheumatoid arthritis	1.25E−09
hsa04151	PI3K-Akt signaling pathway	1.55E−09

AGE-RAGE = advanced glycosylation end-products to the receptor for advanced glycosylation end-products, HIF1 = hypoxia-inducible factor-1, IL-17 = interleukin-17, KEGG = Kyoto encyclopedia of genes and genomes, PI3K-Akt = phosphoinositide 3-kinase to akt serine/threonine kinase, Th17 = helper T cells 17, TNF-α = tumor necrosis factor.

### 3.6. Molecular docking results

**Figure 8.**
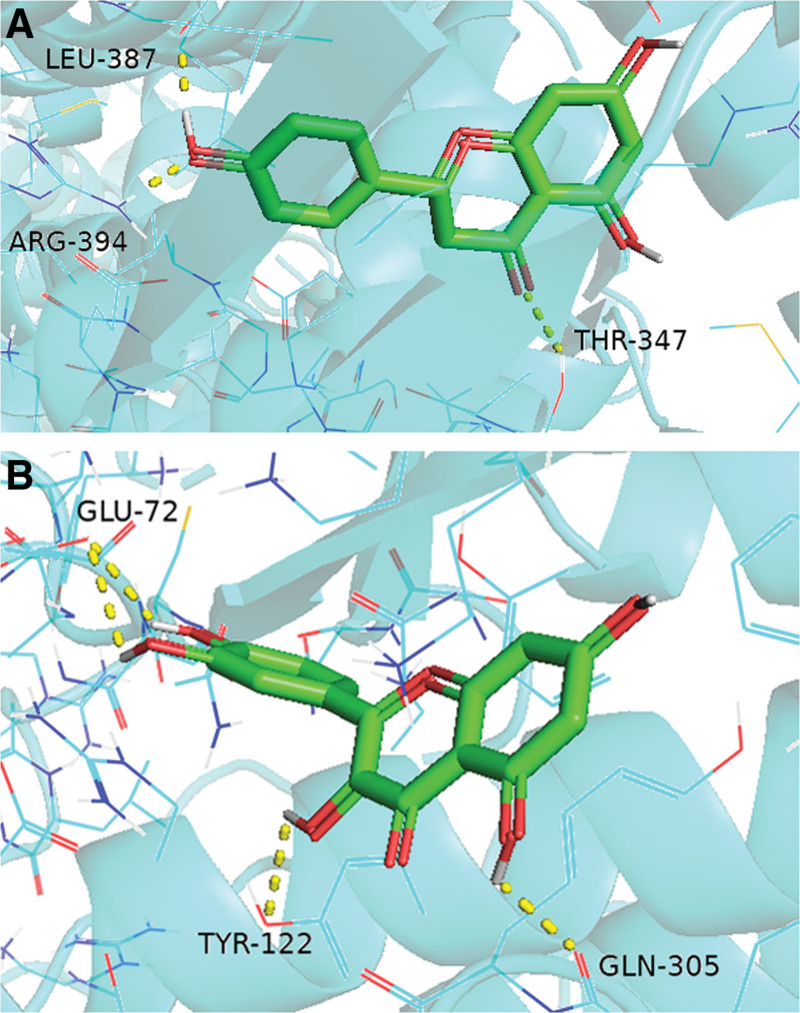
Molecular docking diagram. (A) ESR1-naringenin, (B) STAT3-quercetin.

We docked 3 key active ingredients with targets (AKT1, MYC, HSP90AA1, HIF1A, ESR1, TP53, and STAT3). If the binding energy between ligand and receptor is ≤−5 kcal·mol^−1^, it indicates that a small molecule ligand can bind to a protein receptor efficiently. The binding ability increased with lower binding energy. The results displayed that the binding energies of key active ingredients and core target docking were ≤−5 kcal·mol^−1^ (Table 5). To provide visual insights into the binding activities, we employed PyMOL software to visualize the molecular docking results of naringenin-ESR1 and quercetin-STAT3, as these interactions exhibited the strongest binding activity (Fig. 8).

**Table 5:** Binding energy of active ingredients to core targets.

Compounds	Binding energy/kcal·mol^−1^						
	AKT1	MYC	HSP90AA1	HIF1A	ESR1	TP53	STAT3
Quercetin	−6.3	−6.9	−7.6	−8	−8.4	−6.6	−8.5
Kaempferol	−6.1	−7.1	−7.5	−8	−8	−6.7	−8.1
Naringenin	−6.2	−7	−7.8	−7	−8.5	−6.6	−8.4

AKT1 = serine/threonine kinase 1; ESR1 = estrogen receptor 1; HIF1A = hypoxia-inducible factor-1alpha; HSP90AA1 = heat shock protein 90 alpha family class A member 1; MYC = myelocytomatosis; STAT3 = signal transducer and activator of transcription 3; TP53 = cell tumor antigen p53.

## 4. Discussion

Current epidemiological studies have discovered that the rapid development of economic levels has led to changes in people’s eating habits and pace of life, and the prevalence of NAFLD has increased yearly.^[17]^ The NAFLD pathogenesis is unclear, primarily related to insulin resistance, abnormal lipid metabolism, oxidative stress, inflammatory response, and immune regulation imbalance.^[18]^ TCM prescription is composed of different Chinese medicines with the distinct benefits of multi-ingredient, multi-target, and multi-pathway and helps treat NAFLD with complex pathogenesis. The application of network pharmacology represents a cutting-edge approach in elucidating the mechanisms of TCM prescription interventions in diseases. Therefore, this study used it to explore the mechanism of LGZGD in improving NAFLD, to find a new target for drug treatment of NAFLD, and provide a reference for improving the clinical efficacy and drug safety.

According to network pharmacological analysis, LGZGD may treat NAFLD via quercetin, kaempferol, naringenin, and other major active ingredients. Molecular docking results also exhibited that them had good binding ability with multiple key targets. Recent studies have increasingly highlighted the potential of dietary polyphenolic compounds as therapeutic agents or nutritional supplements for various liver diseases.^[19]^ The main groups of polyphenols include flavonoids, phenolic acids, lignands, stilbenes, and curcuminoids.^[20]^ Flavonoids, being the most abundantly occurring and extensively studied among the polyphenols, demonstrate considerable potential in treating NAFLD due to their inherent safety, widespread availability, and diverse biological activities.^[21–23]^ The flavonoids identified in this study, namely, quercetin, kaempferol, and naringenin, offer promising advantages in NAFLD treatment through various mechanisms, which is consistent with findings from other studies.^[24]^ Many in vivo and in vitro experimental evidence have identified various molecular mechanisms of quercetin in preventing the occurrence and progress of NAFLD, including antioxidant stress, anti-inflammatory, improving fatty acid metabolism, and regulating gut microbiota and bile acid metabolism.^[25]^ Quercetin has therapeutic potential for NAFLD-related learning and memory disorders by regulating glucose and lipid metabolic dysfunction, balancing the protein expression of synaptic plasticity markers, and triggering receptors expressed on myeloid cells-1/2 in the hippocampus.^[26]^ Quercetin can prevent NAFLD by regulating mitochondrial ROS-mediated ferroptosis and improving liver lipid toxicity and accumulation.^[27]^ Through multi-omics research, Lu^[28]^found that kaempferol can improve lipid metabolism, oxidative reaction and inflammation in treating nonalcoholic steatohepatitis (NASH) mice. Kaempferol and kaempferoside can reduce lipid accumulation and oxidative stress in HepG2 cells induced by oleic acid, potentially benefiting NAFLD treatment.^[29]^ Naringenin inhibits NLR family proteins containing Pyrin-related domain 3/nuclear factor-kappaB Signaling pathway alleviates inflammation in the mice liver to prevent NAFLD.^[30]^ Naringenin alleviates NAFLD by directly and indirectly activating adenosine monophosphate-activated protein kinase to increase energy expenditure and regulate autophagy.^[31]^ Naeini F^[32]^ discovered that naringenin could regulate energy balance, glucose and lipid metabolism, inflammation, and oxidative stress and reduce triglyceride accumulation in hepatocytes. Therefore, LGZGD may play the role of antioxidant stress, anti-inflammatory, improving lipid metabolism, and regulating intestinal microbiota in treating NAFLD via quercetin, kaempferol, naringenin, and other active ingredients.

According to the network diagram of “TCM-active ingredients-potential targets” and PPI network topology analysis, the key potential targets are AKT1, MYC, HSP90AA1, HIF1A, ESR1, TP53, and STAT3. Genetic variations in AKT1 have been associated with NAFLD risk.^[33]^ Forkhead box protein O1 (FoxO1) is the main target of insulin action, possibly leading to lipid and glucose metabolism disorders after being activated.^[34]^ Adiponectin can improve NAFLD by inhibiting the FoxO1 expression via AKT1/FoxO1 signaling pathway.^[35]^ In a study by Jiang,^[36]^ that an NAFLD mouse model expressed lower levels of AKT1 and higher interleukin-6, mitogen-activated protein kinase 1, caspase 3, p53, and vascular Endothelial Growth Factor A compared to those observed in the normal group. MYC is crucial for cell proliferation. C-MYC signal transduction is associated with NAFLD-related fibrosis.^[37]^ The specific reduction of MYC in the mouse intestine can improve HFD-induced obesity, insulin resistance, liver steatosis, and steatohepatitis.^[38]^ Peroxisome proliferator-activated receptor-γ (PPARγ) is involved in the NAFLD pathogenesis via adipogenesis, insulin resistance, inflammation, and oxidative stress.^[39]^ HSP90AA1 is a common subtype of HSP90. HSP90 can regulate the activity of PPARγ in NAFLD, which is of great significance for treating NAFLD.^[40]^ HIF-1α is a key regulatory factor that plays an active role in hypoxia. HIF-1α can increases the expression of interleukin-1 beta (IL-1β) by mediating autophagy injury of macrophages, thus promoting the occurrence of NASH.^[41]^ Hypoxia can induce the upregulation of HIF-1α, activating hepatic stellate cells, which ultimately results in hepatic fibrosis.^[42]^ Yexiaodan^[43]^ discovered that inhibiting the HIF-1α/PPARγ signaling pathway can decrease inflammation and steatosis. The ESR1 expression is lower in men than women, but ESR1 knockout will cause insulin resistance phenotype, presenting obesity, glucose intolerance, liver steatosis, and metabolic inflammation of adipose tissue and liver.^[44]^ P53 regulates various metabolic pathways, including lipid metabolism, and regulates lipid synthesis, transport, and decomposition via multiple pathways.^[45]^ Upregulation of the p53 pathway can accelerate NAFLD development by promoting hepatocyte senescence and apoptosis.^[46]^ Silencing functional p53 can alleviate NAFLD by promoting autophagy induced by high mobility group box-1 protein.^[47]^ In addition to its involvement in cell proliferation, STAT3 is closely associated with liver damage, inflammation, regeneration, and cancer.^[48]^ A significant correlation exists between STAT3 activation and the severity of hepatic fibrosis in patients with NAFLD.^[49]^ It is speculated that LGZGD can intervene in insulin resistance, inflammation, oxidative stress, and lipid metabolism of NAFLD via the above targets to achieve the therapeutic purpose.

LGZGD is primarily associated with PI3K/Akt, IL-17, TNF, Th17 cell differentiation, HIF-1, and TLR signaling pathways in treating NAFLD. PI3K/Akt signaling pathway is the key pathway of insulin information transmission, and its signal transmission disorder easily leads to insulin resistance, assisting in the development of NAFLD.^[50]^ Activation of PI3K/Akt-nuclear factor E2 related factor 2-antioxidant response element pathway can improve liver oxidative stress and inflammatory response.^[51]^ By stimulating autophagy, the inhibition of the PI3K/Akt/mammalian target of the rapamycin signaling pathway can alleviate lipid accumulation, inflammation and NAFLD.^[52]^ Th17 cells are an important subset of CD4^+^ lymphocytes that participate in various inflammatory and immune diseases and can induce epithelial cells to secrete interleukin-6, IL-17, TNF- α, and other proinflammatory factors, thus promoting hepatocyte inflammation and steatosis.^[53]^ As the primary effector of Th17 cells, IL-17 deficiency can interfere with intestinal microbiota composition in methionine and choline-deficient diet-fed mice, such as increased pathogenic bacteria abundance and decreased probiotics, aggravate the liver steatosis accumulation and promote the NAFLD/NASH progress.^[54]^ Among the many pro-inflammatory factors that contribute to the pathogenesis of NASH, TNF- α has a crucial role in many aspects of NASH progress.^[55]^ HIF-1 mediates liver fibrosis development in the NAFLD mouse model.^[56]^ TLR is widely expressed in hepatocytes, Kupffer cells, and hepatic satellite cells.^[57]^ The majority of TLRs induce pro-inflammatory and pro-fibrotic cytokines, participating in the progression of NAFLD.^[58]^ Palmitic acid-induced hepatocyte lipotoxicity is mediated by the activation of TLR4 inositol-dependent kinase 1α pathway.^[59]^ Conversely, inhibition of TLR2 improves lipopolysaccharide-induced lipid accumulation and toxicity in hepatocytes.^[60]^

Docking experiments confirmed that quercetin, kaempferol, and naringenin exhibit strong binding affinity with core targets (AKT1, MYC, HSP90AA1, HIF1A, ESR1, TP53, and STAT3), with binding energies less than −5 kcal·mol^-1^. This suggests that the therapeutic potential of LGZGD in treating NAFLD may be attributed to the efficacy of these 3 key active ingredients.

## 5. Conclusion

This study concludes that LGZGD may have multi-ingredient, multi-target, and multi-pathway effects in treating NAFLD, possibly playing a therapeutic role by improving insulin resistance, antioxidant stress, anti-inflammatory, lipid metabolism, and other mechanisms. The results exhibited that quercetin, kaempferol, and naringenin were the primary active compounds in LGZGD. Meanwhile, AKT1, MYC, HSP90AA1, HIF1A, ESR1, TP53, and STAT3 are the potential therapeutic targets of LGZGD in treating NAFLD. LGZGD can treat NAFLD by regulating PI3K/Akt, IL-17, TNF, and other signaling pathways. This study provides a new idea, direction, and reference for the research of LGZGD for treating NAFLD and a theoretical basis for developing new drugs for LGZGD.

## Author contributions

**Data curation:** Songlin Gao, Liuting Wei, Peng Zhang, Fei Liang.

**Formal analysis:** Songlin Gao, Liuting Wei.

**Funding acquisition:** Guihua Huang.

**Methodology:** Songlin Gao, Liuting Wei.

**Project administration:** Guihua Huang.

**Software:** Yan Qin.

**Supervision:** Guihua Huang.

**Visualization:** Liuting Wei, Yan Qin, Tingwei Quan.

**Writing – original draft:** Songlin Gao.

**Writing – review & editing:** Guihua Huang.
